# Effect of transcutaneous electrical stimulation treatment on lower urinary tract symptoms after class III radical hysterectomy in cervical cancer patients: study protocol for a multicentre, randomized controlled trial

**DOI:** 10.1186/s12885-017-3387-1

**Published:** 2017-06-15

**Authors:** Xiu-Li Sun, Hai-Bo Wang, Zhi-Qi Wang, Ting-ting Cao, Xin Yang, Jing-Song Han, Yang-feng Wu, Kathleen H. Reilly, Jian-Liu Wang

**Affiliations:** 1 Department of Obstetrics and Gynecology, Peking University People’s Hospital, No.11 Xizhimen South Street, Xicheng Dist, Beijing, 100044 China; 2 Peking University Clinical Research Institute, Xueyuan Rd 38#, Haidian Dist, Beijing, 100191 China; 3 Peking University Third Hospital, Huayuan North Rd 49#, Haidian Dist, Beijing, 100191 China; 4 The George Institute for Global Health at Peking University Health Science Center, Beijing, 100191 China; 5 Department of Epidemiology and Biostatistics, Peking University School of Public Health, Beijing, 100191 China; 6 New York City, NY USA

**Keywords:** Transcutaneous electrical stimulation, Cervical cancer, Lower urinary tract symptoms, Class III radical hysterectomy

## Abstract

**Background:**

Class III radical hysterectomy (RH III)_plus pelvic lymphadenectomy is the standard surgery for early stage cervical cancer (CC) patients, the 5 year survival rate is about 90%, but pelvic floor disorders especially bladder dysfunction are common due to damaged vessels and nerve fibers following surgery. Transcutaneous electrical stimulation (TENS) treatment has been used to treat bladder disorders for many years, but its effect on cervical cancer patients, the best treatment time point and stimulated protocol, had never been assessed. The aim of this study is to investigate the efficacy of TENS treatment on lower urinary tract symptoms (LUTS) after RH III in CC patients.

**Methods/Design:**

The study will be conducted as a clinical, multicentre, randomised controlled trial with balanced randomisation (1:1). The planned sample size is 208 participants (at 1:1 ratio, 104 subjects in each group). At 5–7 days after RH III, patients are screened according to operative and pathological findings. Enrolled participants are randomised into an intervention group (TENS plus conventional clinical care) or control group (conventional clinical care), with stratification by menopausal status (menopause vs. non-menopause) and surgical modality (laparoscopic RH or abdominal RH). Participants in both groups will be followed up at 14 days, 21 days, 28 days, 3 months, 6 months, 12 months, 18 months and 24 months after surgery. The primary endpoint is improvement rate of urination function which is defined as recovery (residual urine ≤50 ml) or improvement (residual urine 50–100 ml). Secondary endpoints include urodynamic parameter, urinary incontinence, anorectal function, pelvic function, quality of life (QOL), disease-free survival and adverse events. Primary endpoint analyses will be carried out by Cochran-Mantel-Haenszel tests taking into center effect.

**Discussion:**

To our knowledge this is the first trial to investigate the effect of TENS treatment on bladder function recovery after RH III among CC patients. This study will provide new information on TENS efficacy for bladder function recovery. Once confirmed, it may help to provide a new, non-invisive treatment for those postoperative CC patients with poor pelvic function, which would help improve their quality of life.

**Trial registration:**

The study is registered to Clinical Trials.gov (NCT02492542) on June 25, 2015.

## Background

Cervical cancer is one of the most commonly diagnosed malignant tumors among women all over the world [1]. There are an estimated 131.5 thousand newly diagnosed cervical cancer cases per year in China, accounting for 28.8% of the world’s cases. In all, 30% of cervical cancer patients are younger than 30 years [2]. With the widespread availability of cervical cancer screening, the proportion of early stage cervical cancer is increasing year by year. The standard treatment for patients with early stage cervical cancer is class III radical hysterectomy plus pelvic lymphadenectomy, with chemotherapy and/or radiotherapy supplemented as needed. The 5 year survival rate after treatment can reach as high as 90% [2]. Pelvic vessels, autonomic nerve fibers and ligaments could be affected by the resection of anterior, lateral and posterior parametrium and vaginal cuff. The unintended interruption may result in pelvic floor dysfunction including disorders of lower urinary tract, defecation disorders and sexual dysfunction. Though the cancer could be cured by the surgery, many patients may suffer from decreased quality of life due to the poor pelvic function following radical hysterectomy.

It has been reported that 70%–85% cervical cancer patients suffer from bladder dysfunction following radical hysterectomy [3, 4]. The main symptom of short-term bladder dysfunction is urinary retention (UR) while the long-term postoperative bladder disorder is urinary incontinence [5, 6]. Usually the time of indwelling urinary catheter after class III radical hysterectomy is 2 weeks, but it lasts up to 1 month in about 15% patients and even more than half a year in 3% patients because of chronic urinary retention [6, 7].

In clinical practice, doctors have tried many therapeutic methods to prevent and manage UR in patients who underwent radical hysterectomy. Among these methods, early postoperative bladder training is most commonly used, the urinary catheter is clamped and unclamped every 2–4 h to let the bladder fill and empty, aiming to exercise the detrusor contraction ability. But a recent randomized trail showed that this bladder training did not reduce the rate of UR or re-admission for bladder catheterization [8]. Some Chinese doctors use acupuncture to treat UR after radical hysterectomy with potential efficacy [9], but more studies are needed to prove its effects due to limited sample size and poor study design, moreover, acupuncture is difficult to apply, in general, due to the specialized technique. For those with chronic UR, clean intermittent catheterization or even suprapubic cystostomy is needed which are associated with frequent urinary tract infection [10].

Recently, electrical stimulation has been used to treat bladder disorders, especially for urinary retention and incontinence and the efficacy is promising [11, 12]. The electrical stimulations mostly used were transcutaneous electrical stimulation (TENS), percutaneous tibal nerve stimulation (PTNS) and sacral neuromodulation (SNM). TENS is noninvasive, easy performed and low cost compared to the other treatment options. Electrical stimulation of the sensory fibers of the pudendal nerve with low frequency (2 ~ 5HZ) evoked bladder contraction, resulting in increasing voiding efficiency, even in acute spinal transection abolished in animal models [13]. Few trials have explored the electrical stimulation for treatment of VR in cervical cancer patients [14–16], and these trails had small numbers of participants. We therefore conducted this RCT trail to verify if early postoperative TENS treatment can reduce the rate of VR in cervical cancer patients who underwent class III radical hysterectomy.

## Methods and design

### Trial design and setting

**Fig. 1:**
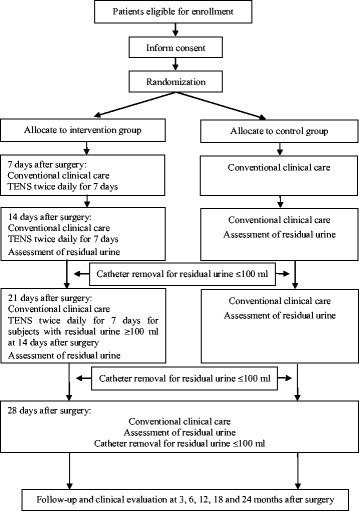
Study flow chart

The trial is conducted as a clinical, multicentre, randomised controlled trial with balanced randomisation (1:1) to examine the efficacy of transcutaneous electrical stimulation (TENS) treatment on the recovery of urinary function among postoperative patients with early cervical cancer. After class III radical hysterectomy, participants with early cervical cancer (International Federation of Gynecology and Obstetrics [FIGO], stage Ia2, Ib1 and IIA1) will be randomly assigned to an intervention or control group. Participants will be stratified by menopausal status and surgical modality. Subjects will be recruited continuously by oncologists from gynecology departments in nine hospitals of Beijing city: Peking University People’s Hospital; Peking University First Hospital; Peking University Third Hospital; Cancer Institute and Hospital, Chinese Academy of Medical Sciences; Peking University Cancer Hospital & Institute; The General Hospital of the People’s Liberation Army; Beijing Obstetrics and Gynecology Hospital, Capital Medical University; Beijing Chaoyang Hospital, Capital Medical University; and Beijing Hospital. Figure 1 illustrates the flow diagram of the study for both the intervention and control groups.

### Participants

#### Inclusion criteria

Participants satisfying the following inclusion criteria were enrolled: (1) aged 18–60 years; (2) diagnosed with clinical early cervical squamous cell carcinoma (FIGO stage Ia2, Ib1 and IIA1); (3) received the surgery of class III radical hysterectomy; (4) negative resection margins indicated by biopsy and without distant metastasis as follows: lymph node (−), depth of myometrial invasion <1/2, lymphovascular space invasion (−), resection margin (−) and with G1/G2 histological differentiation; and (5) willing to participate in the study and sign informed consent.

#### Exclusion criteria

If one of the following criteria is met, patients will be excluded from the study: (1) participants who received neoadjuvant chemotherapy, radiotherapy or chemoradiotherapy before and after operation; (2) received nerve sparing surgery; (3) urinary system injury during a gynecologic operation; (4) diagnosed with ≥ stage II pelvic organ prolapse before operation; (5) preoperative moderate to severe stress urinary incontinence (pad weight testing ≥10 g); (6) preoperative urinary retention; (7) preoperative serious functional constipation or defecation disorder; and (8) participant’s inability to fully comply with study protocol due to uncontrolled epilepsy, central nervous system diseases or psychosis.

### Randomisation and blinding

Central randomisation via interactive web response system will be carried out by Peking University Clinical Research Institute, which is independent of the trial administration office. The allocation sequence is computer-generated 1:1 with dynamic randomisation system using minimization. Randomisation is conducted with varying block size and will be stratified by menopausal status (menopause vs. non-menopause) and surgical modality (laparoscopic radical hysterectomy or abdominal radical hysterectomy). Intervention assignment cannot be blinded for the participants and investigators due to device stimulation and blank control.

### Intervention

All participants who are assigned to the intervention group will receive TENS treatment on the basis of conventional clinical care. The stimulation treatment starts 7 days (±2 days) after radical hysterectomy. Participants receive TENS treatment twice daily for 14 days (28 treatment sessions), and those patients with residual urine >100 ml at 14 days post-operation will take an additional 7 day stimulation treatment (14 treatment sessions). Patients are placed in a supine decubital position. The electric parameters used in each session are homogeneous: current type: functional electrical stimulation; frequency: 1/4/1 Hz; pulse duration: 270/230/270 μs; time: 30 min; surface electrode: 50*50 MM adhesive electrode; stimulation intensity: maximal level tolerable without pain, usually less than 100 mA. One electrode is placed in the S3 region and the other one is crossed on the skin and fixed at the bladder area.

All subjects who are assigned to the control group will receive only conventional clinical care. With the exception of TENS, intervention and control groups have the same study procedures throughout the entire study. The catheter is removed while residual urine is less than 100 ml at 14 days, 21 days or 28 days after surgery. Catheter removal timing is determined according to the recovery of participants if residual urine is >100 ml at 28 days post-operation.

### Initial screening, assessment and follow-up

After providing informed consent, participants are asked standardized questions about their demographic characteristics, socioeconomic status and medical history. Clinical data is extracted from the medical records regarding primary tumor, time of primary diagnosis, surgical operation, physical examination, laboratory testing, gynecological examination and imaging examination (Table 1).

**Table 1:** Baseline screening, assessment, and follow-up schedule

	At baseline	Time after surgery								Early withdrawal
		14 days	21 days	28 days	3 months	6 months	12 months	18 months	24 months	
Follow-up time window (days)		±2	±2	±2	±7	±7	±7	±7	±7	
Informed consent	●									
Demographic characteristics	●									
Socioeconomic status	●									
Medical history	●									
Information on tumor clinical evaluation and surgical operation	●									
Physical examination	●									
Blood routine examination	●				●	●	●	●	●	
Blood clotting function	●									
Routine urine test	●				●	●	●	●	●	
Routine stool test	●				●	●	●	●	●	
Blood biochemistry	●				●	●	●	●	●	
Hormone testing^a^	●				●	●	●	●	●	●
Tumor biomarkers^b^	●				●	●	●	●	●	●
Gynecological examination^c^	●				●	●	●	●	●	●
Abdominal/pelvic CT/MRI	●	▲								
Chess X-ray	●					●	●	●	●	●
Randomization	●									
Assessment of residual urine^d^		●	●	●	●	●	●			
Uroflowmetry				●	●					
Urodynamic tests				●						
Anorectal function evaluation				●						
Pelvic floor functional testing				●	●					
EQ-5D^e^					●	●	●		●	
PISQ-12^f^						●	●		●	
PFDI-20^g^					●	●	●		●	
ICIQ/OABss^h^					●	●	●		●	
Adverse events	●				●	●	●	●	●	●

●: Indicates mandatory items

▲: Investigator will decide whether to perform the testi according to clinical signs or clinical evaluation

^a^Hormone testing includes estradiol, progesterone, testosterone, prolactin, follotropin and luteinizing hormone

^b^Tumor biomarkers includes squamous cell carcinoma antigen, CA125, CA199 and carcinoembryonic antigen

^c^Gynecological examination includes Human Papillomavirus and Thinprep Cytological Testing

^d^Residual urine is evaluated by B ultrasound

^e^EQ-5D: European Quality of Life-5 Dimensions

^f^PISQ-12: Prolapse/Urinary Incontinence Sexual Questionnaire short form

^g^PFDI-20: Pelvic Floor Distress Inventory

^h^ICIQ/OABss: International Consultation on Incontinence Questionnaire/Overactive Bladder Symptom Score

All the enrolled subjects will be followed up at 14 days, 21 days, 28 days, 3 months, 6 months, 12 months, 18 months and 24 months after the surgical operation. Residual urine following self-urination is evaluated by B ultrasound at postoperative 14 days, 21 days, 28 days, 3 months, 6 months and 12 months. Uroflowmetry, urodynamic parameter, anorectal function and pelvic floor function are assessed at postoperative 28 days; in addition, uroflowmetry and pelvic floor function are evaluated at 3 months after surgery. Quality of life (QOL) is evaluated by European Quality of Life-5 Dimensions (EQ-5D), Prolapse/Urinary Incontinence Sexual Questionnaire short form (PISQ-12), Pelvic Floor Distress Inventory (PFDI-20) and International Consultation on Incontinence Questionnaire/Overactive Bladder Symptom Score (ICIQ/OABss). All QoL related questionnaires are required to be completed during the follow-up at 3 months, 6 months, 12 months and 24 months after surgery, except PISQ-12 at 3 months. During the follow-up at 3 months, 6 months, 12 months, 18 months and 24 months after surgery, information related with laboratory testing, gynecological examination, chest X-ray and adverse events will be collected and assessed.

### Study endpoints

The primary endpoint is improvement rate of urination function. Based on self-urination status and residual urine evaluated by B ultrasound following catheter removal and self-urination, efficacy is classified into three levels: 1) recovery: automatic micturition is achieved by patients with residual urine ≤50 ml; 2) improvement: automatic micturition is achieved by patients with residual urine 50–100 ml; 3) invalid: automatic micturition is not achieved, or automatic micturition is achieved but with residual urine ≥100 ml. Improvement rate is calculated as follows: (the number of recovery participants + the number of improvement participants)/the total number of enrolled participants *100%.

Secondary endpoints are: (1) urodynamic parameter, including maximal uroflowmetry, micturition curve, bladder sensation, detrusor muscle stability, detrusor muscle function of contraction, stress urinary incontinence, urinary retention and lower urinary tract obstruction; (2) urinary incontinence evaluated by 1 h pad test is defined as follows: <1 g continence, 1–10 g mild incontinence, 11-30 g moderate incontinence, 31-50 g severe incontinence, >50 g extremely severe incontinence; (3) anorectal function, including rectal sensation, rectal maximum volume, reflex of rectal anal internal sphincter, reflex of rectal anal external sphincter and balloon expulsion testing; (4) pelvic function, including muscle strength of type I and type II muscle fiber of pelvic muscle, fatigue of pelvic muscle, vaginal dynamic pressure and neural reflex; (5) QOL evaluated by EQ-5D, PISQ-12, PFDI-20 and ICIQ/OABss; (6) overall survival (OS), calculated as the time from initial diagnosis to death from any cause or the date of last follow-up for surviving subjects; (7) disease-free survival (DFS), defined as the period from the date of initial diagnosis to the date of recurrence, metastasis, death due to any cause or the date of last follow-up for surviving subjects; (8) adverse events.

### Sample size consideration

We expect improvement rate of urination function 70% in the intervention group and 50% in the control group. Based on a difference of 20% between groups on the primary outcome, assuming 10% drop-out rate, a total of 208 participants (at 1:1 ratio, 104 subjects in each group) are required to provide 80% power, with the use of two-sided significance level of 0.05.

### Statistical analysis

Analyses will be made using SAS statistical software (version 9.3) by a statistician in Peking University Clinical Research Institute. Data will be analyzed according to the “intent-to-treat” principle. A two-sided significance level of 5% will be used for all analyses.

Descriptive statistics will be used to summarize demographic and clinical characteristics of participants randomised to the intervention and control groups. The difference between two groups on demographic characteristics, socioeconomic status, clinical characteristics and follow-up retention rate will compared using t-tests (or Wilcoxon rank sum test) and chi-square test/Fisher’s exact test as appropriate.

Primary outcome (improvement rate of urination function in 1 year) analyses will be carried out by Cochran-Mantel-Haenszel tests taking into center effect.

The difference in urodynamic parameter, anorectal function and pelvic function between intervention and control groups will be determined using t-tests or Wilcoxon rank sum tests as appropriate. The severity of urinary incontinence will be analyzed using the Wilcoxon rank sum test. The differences in QOL (evaluated by EQ-5D, PISQ-12, PFDI-20 and ICIQ/OABss) between intervention and control group will be determined using repeated measure analysis of variance (ANOVA). DFS and OS survival curves will be calculated using the Kaplan-Meier method, and compared using the log-rank test. Cox proportional hazards models will also be used to calculate hazard ratio and 95% confidence interval, with other risk factors (such as cancer stage and tumor localization) as covariates.

The incidence of adverse events between 2 groups will be compared with the chi-square test/Fisher’s exact test. Descriptive statistics will be used to summarize incidence rates of adverse events and serious adverse events.

## Discussion

Lower urinary tract symptoms (LUTS) are usual complications of radical hysterectomy among cervical cancer patients. Among these symptoms, UR, dysuria, loss of bladder sensory and urinary incontinence are most common [17]. Some patients even need suprapubic cystostomy resulting in poor life quality. As an non-invasive treatment method, TENS has been used to treat pelvic floor disorders including bladder dysfunction and has been proven to be effective [11]. TENS modifies the electric impulses of nerve fibers, promotes blood circulation of pelvic organs, thus might help the recovery of bladder sensitivity and increase the contractility of the bladder detrusor.

But by now only few studies have been carried out using electrical stimulation treatment on cancer patients [14–16]. Among these few studies, a trial conducted by Gianna Mariotti [15] had the highest quality based on RCT design. In this study, 60 patients who underwent radical prostatectomy were prospectively randomised to treatment group and control groups. Patients in the treatment group received electrical stimulation and biofeedback from the 7th day postoperative. The treatment was performed twice a week for 6 weeks, patients were followed up at 1, 2, 3, 4, 5 and 6 months. The results showed that treatment with biofeedback and pelvic floor electrical stimulation had a significant positive impact on the early recovery of urinary continence after radical prostatectomy. A previous study conducted by our research team showed that transcutaneous low frequency electrical stimulation from the 11th day after radical hysterectomy can reduce the residual urine volume and shorten the time of indwelling urinary catheter [16].

No research has mentioned the safety of electrical stimulation on cancer patients except our in vitro study, which indicated that electrical stimulation had no effect on cell proliferation, invasion and immigration capacity [18]. Due to the safety uncertainty of TENS on cancer patients, the trial presented here does not include all cervical cancer patients, those who had a high risk of recurrence and might need further ajuvant chemotherapy and/or radiaotherapy will be excluded. Thus all participants are with early cervical cancer (stage Ia2, Ib1 and IIA1, FIGO stage), no other risk factors differed by pathologic diagnosis.

To our knowledge this is the first trial to investigate the effect of TENS treatment on bladder function recovery after class III radical hysterectomy among cervical cancer patients. If our research findings are positive, we may provide a new, non-invasive treatment method for those postoperative cervical cancer patients with poor pelvic function, to improve their quality of life.

## Abbreviations

LUTS: Lower urinary tract symptoms
PTNS: Percutaneous tibal nerve stimulation
QOL: Quality of life
SNM: Sacral neuromodulation
TENS: Transcutaneous electrical stimulation
UR: Urinary retention.
